# Protozoan Parasites of Iranian Freshwater Fishes: Review, Composition, Classification, and Modeling Distribution

**DOI:** 10.3390/pathogens12050651

**Published:** 2023-04-27

**Authors:** Maryam Barzegar, Mehdi Raissy, Shokoofeh Shamsi

**Affiliations:** 1 Faculty of Veterinary Medicine, Garmsar Branch, Islamic Azad University, Garmsar 3581631167, Iran; 2 Department of Aquatic Animal Health and Disease, Faculty of Veterinary Medicine, Shahrekord Branch, Islamic Azad University, Shahrekord 8813733395, Iran; 3 Gulbali Institute and School of Agricultural, Environmental and Veterinary Sciences, Charles Sturt University, Wagga Wagga, NSW 2650, Australia

**Keywords:** protozoan parasites, freshwater fish, checklist, geographical distribution, Iran

## Abstract

This article investigates the occurrence and distribution of parasitic protozoa of Iranian freshwater fishes (both farmed and wild). Our search shows 26 known parasitic protozoan species were recorded from 52 freshwater fish species across different ecoregions of Iran. Most of these fish are edible. While none of the identified protozoan parasites are of zoonotic importance, our study does not exclude presence of zoonotic species in Iranian fishes. Present data suggest the northern and western regions of the country are the main macrohabitat of protozoa (35 parasitic records reported), with the greatest concentration of parasitic protozoa occurring in the Urmia basin in Iran’s northwest. The clustered distribution pattern of protozoa among freshwater fish was also more evident in the northern and western parts of the country. The gills and skin were the most infected microhabitats for parasitic protozoa. The highest number of parasites was observed in the fish family Cyprinidae with nine species found in the native fish, *Capoeta capoeta*. The most diverse host range was observed in the holotrich ciliate, *Ichthyophthirius multifiliis* isolated from 46 cyprinid species in 39 different locations. However, due to the great richness of fish and extreme habitat diversity, parts of the parasite fauna of Iranian freshwater fish are still poorly understood. Furthermore, current and future changes in climate and environmental parameters, and anthropogenic interventions are likely to affect fish hosts and their parasites.

## 1. Introduction

Protozoans are one of the major threats to fish health, causing diseases in both farming and wild systems [1]. Parasitic protozoa, particularly those with direct lifecycles and broad host specificity, can easily transmit within fish populations [2]. Parasitic invasion can adversely affect growth rate, cause weight loss, and suppress reproductive activities. Severe infection can lead to extant mortality and massive destruction of fish stock [3,4,5]. Some protozoa are ectoparasites that inhabit skin, fins, and/or gills, while others invade internal organs, such as the intestine.

Research on protozoan parasites of freshwater fish in Iran has been limited. A few studies, mainly on ectoparasitic protozoa, have examined the prevalence, intensity, histopathology, taxonomy, and systematic classification of protozoan parasites [6,7,8,9,10,11,12]. Protozoan parasites infecting Iranian freshwater fish were first reported by Jalali [7], who studied the pathogenesis and diagnosis of common parasites, discussing the interrelationship between fish and parasites extensively. A subsequent checklist [10] included 23 protozoan parasite species from 30 fish hosts, but nomenclature of the protozoa taxa and fish hosts reported in Iran was outdated and contained several errors. In many cases, the parasite life stage and precise names of localities were missed. Therefore, the present study aims to update the classification and nomenclature of protozoan parasites and their hosts, and correct possible misidentifications and misspellings made in previous studies. This paper presents a detailed list of parasites found in each host, and a spatial distribution map of the drainage basin for the localities where infected freshwater fishes have been caught. Different global change scenarios are also presented in by modeling past and present spatial distributions of freshwater fish protozoa in Iran.

## 2. Materials and Methods

### 2.1. Study Area

Data used in the present study include lakes, wetlands, reservoirs, rivers, streams, estuaries, bays, springs, and aquaculture facilities throughout Iran, lying between latitudes 24° and 40° N, and longitudes 44° and 64° E. The recorded geographical features are distributed throughout 16 endorheic drainage basins, including Bejestan, Caspian Sea, Dasht-e Kavir, Dasht-e Lut, Isfahan, Hamun-e Mashkid, Hamun-e Jaz Murian, Kor River, Lake Maharlu, Lake Urmia, Namak Lake, Sirjan, Sistan, Hari (Tedzhen) River, and Kerman-Na’in.

### 2.2. Search Strategy

**Figure 1:**
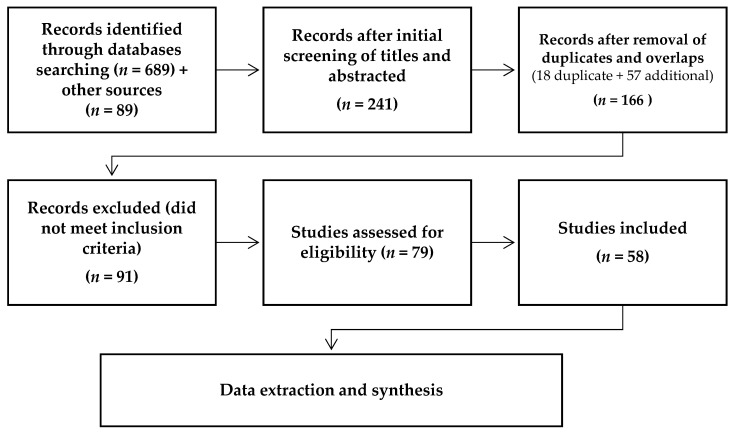
Flowchart of review process.

Figure 1 summarizes the research strategy of this study. Published records on lentic and lotic environments from 1981 to 2022, for native and introduced fish species in wild and farmed systems, were included. Zoological Record, Biological Abstracts, Fisheries Abstracts, Web of Knowledge, Scopus, Google Scholar, the Iranian Research Institute for Information Science and Technology (IranDoc), Scientific Information Database (SID), open access databases, and the research repository of Aquadocs were searched for the following words: “fish” or “protozoa” or “Iran”. The bibliographies of the articles found through the search were checked for other relevant articles. Inclusion criteria included peer-reviewed published articles, final reports of the research projects conducted by research institutes affiliated with the Ministry of Science, published conference abstracts of congress meetings and seminars where the parasites were reported at the species level, books, and indexed PhD and Master of Science (M.Sc.) dissertations. The exclusion criteria were unpublished records (gray literature) and those with misidentification, available doubts, duplicate documentation, and those where parasites were identified at the genera level. While conference proceedings and scientific reports may be considered gray literature, they were still included, as there is evidence that they can be valuable [13].

### 2.3. Data Extraction

We followed the classification used in previous studies [14,15], the World Register of Marine Species (https://www.marinespecies.org (accessed on 31 January 2023)), and The National Center for Biotechnology Information (https://www.ncbi.nlm.nih.gov (accessed on 31 January 2023)). All articles were independently screened by two reviewers and assessed for eligibility. Data, including taxonomic levels of parasites and hosts, locality, and the source of the report, were recorded in an MS Excel spreadsheet.

Cases dealing with parasite misidentification and duplicates were removed manually by reviewers, records were screened by going through titles, abstracts, and full texts. Articles in doubt were included in the first instance until further discussion and consensus was reached [16].

### 2.4. Spatial Analysis

#### 2.4.1. Occurrence Record

Where geographic point information was unavailable, coordinates of localities where freshwater fish protozoa have been reported were calculated using ArcGIS Desktop (version 10.8, (Esri, Redlands, CA, USA; the American multinational geographic information system (GIS) software). The “Feature to Point” tool, which creates a feature class containing points generated from the representative locations of input features, was used to calculate the centroid coordinates of the features. For line, polygon, or three-dimensional features, the center of gravity/geometric center of a feature was used, which may fall inside or outside the feature. For multipoint features, such as fish farms and hatchery centers, with a collection of individual point locations stored as coordinate pairs, the gravity center was computed using the weighted mean center of all feature parts and was considered a single record in the database. After calculating the centroid coordinates of the features, the repeated records were rarefied into 5 km distances to reduce spatial autocorrelation. A total of 58 occurrence points were extracted to display the spatial distribution map of each natural or artificial geographic feature in Iran.

#### 2.4.2. Protozoan Occurrences Map

To visualize the spatial distribution map of the features (localities where protozoan species of freshwater fishes have been reported), the GIS database for extracted geographic points was overlaid with the layer of the major drainage basins (watershed boundaries) of the country. To determine whether the spatial distribution of the geographical features was spatially random or clustered, the “average nearest neighbor” measurement tool was used. This tool measures the distance between the center of each feature and the centroid site of its nearest neighbors, then averages all these most relative neighbor distances [17]. The average nearest neighbor (*ANN*) ratio is provided as:

(1) $$\mathrm{ANN}=\frac{\bar{D_{O}}}{\bar{D_{E}}}$$

${\bar{D}}_{O}$ is the observed average distance between each location and its nearest neighbor; ${\bar{D}}_{E}$ is the anticipated average distance for the locations provided in an accidental pattern.

(2) $${\bar{D}}_{O}=\frac{\sum_{i=1}^{n}d_{i}}{n}$$

(3) $${\bar{D}}_{E}=\frac{0.5}{\sqrt{n/A}}$$

where *d_i_* corresponds to the distance between location *i* and its nearest neighboring location, *n* equals the total of locations, and *A* is either the area of a minimum enclosing rectangle around all features, or a user-specified area value. The average of the nearest neighbor *z*-score for the statistic is computed as follows:

(4) $$z=\frac{\bar{D_{O}}-\bar{D_{E}}}{\mathrm{SE}}$$

(5) $$\mathrm{SE}=\frac{0.26136}{\sqrt{n^{2}/A}}$$

Subsequently, kernel density estimation (KDE) modeling in ArcGIS Desktop was calculated to determine important hotspots for protozoan species throughout the country. After assessing the bandwidth (radius), weights were calculated for each point within the kernel radius. As a result, the closest points to the center obtained a higher weight and subscribed more to the cells’ total density value. Eventually, the values of the final grid were determined by adding the values of all circle surfaces for each feature [18]. The predicted density for a new (x,y) feature point is calculated as follows:

(6) $$\mathrm{Density}=\frac{1}{{\left(\mathrm{radius}\right)}^{2}}\overset{n}{\underset{i\;=\;1}{\sum }}\left[\frac{3}{\pi }\;\cdot \;{\;\mathrm{species}}_{i}{\left(1\;-\;{\left(\frac{{\mathrm{dist}}_{i}}{\mathrm{radius}}\right)}^{2}\right)}^{2}\right]$$

For *dist_i_* < *radius*

Where:

- *i* = 1, …, *n* are the input points. Only points that are in the radius distance of the (x,y) location come into account.
- *species_i_* is the species field value (in this case, the number of individual protozoan species) of point *i*.
- *dist_i_* is the distance between the (x,y) location and point *i*.

The IDW [19] tool was also used to create the spatial distribution map of the de Martonne (DM) aridity index for Iran, based on the past two-year average (2020–2022). IDW is a deterministic technique for multivariate interpolation with a set of known scattered points, assuming closer points are more similar than farther ones. The given values to unknown points are estimated with a weighted average of the existing values at the known points. Closer points to the center of the estimated cell have more influence or weight in the averaging process [19]. Inverse distance weighted (IDW) is calculated as follows:

(7) $$Z_{j}=\frac{\sum_{i=1}^{n}\frac{Z_{i}}{{\left(h_{\mathrm{ij}\;}+\delta \right)}^{\beta }}}{\sum_{i=1}^{n}\frac{1}{{\left(h_{\mathrm{ij}}+\delta \right)}^{\beta }}}$$

where *Z_j_* is the value of an unknown point, *Z_i_* is the value of a known point, *β* is the weight and *δ* is a correction variable. The separation distance between a known and unknown point, *h_ij_* is determined using the Euclidean method:

(8) $$h_{ij}=\sqrt{{\left(\Delta x\right)}^{2}+{\left(\Delta y\right)}^{2}}$$

where Δ*x* and Δ*y* are the distances between the known point *i* and the unknown point *j* depending on reference axes.

In the present study, precipitation and temperature observation datasets were obtained from the Meteorological Organization of Iran and used as model inputs for modeling on a monthly time scale, while the de Martonne aridity (IDM) was calculated based on the following equation [20]:

IDM = *P*/*T* + 10 (9)

where *P* is the annual amount of precipitation (in millimeters), and *T* is the mean annual air temperature (in degrees Celsius).

The classification of the climatic zones based on the de Martonne climate index is shown in Table 1. Ultimately, the GIS database for geographic points of each locality was overlaid on the spatial distribution map of the DM aridity index for Iran.

### 2.5. Distribution Modeling

#### 2.5.1. Environmental Variables

Nineteen standard bioclimatic variables were downloaded from the latest version (2.1) of the CHELSA dataset (http://chelsa-climate.org (accessed on 1 December 2022) [21]) at a spatial resolution of 30 arc-seconds (~km^2^) for the current period, which is defined as the period from 1981 to 2010. Bioclimatic variables calculated from monthly temperature and precipitation values were generated by interpolating average monthly climate data from weather stations at different spatial resolutions. To avoid multicollinearity in the model, variables that were highly correlated with each other (i.e., showed more than 0.6 Pearson’s correlation coefficient) were removed using the “remove highly correlated variables” tool in SDM Toolbox v2.5 [22]. Therefore, only 12 environmental variables were retained to simulate the distributions of freshwater fish protozoan parasites in Iran (Table 2).

Three topographic variables—elevation, slope, and aspect—were used for the modeling distribution of protozoa. The topographic variable, elevation with a 30 arc-seconds (~km^2^) resolution was derived from the latest version (2.1) of the WorldClim dataset (Fick and Hijmans 2017; http://worldclim.org (accessed on 20 February 2023)); while slope and aspect layers were generated from the elevation raster using the surface analyst tool in ArcMap and added as the variables.

Downscaled future climatic data, with a 30 arc-second (~km^2^) resolution, for two time periods (2050s (2041–2070) and 2080s (2071–2100)) from the latest version of the IPSL climate model were extracted from CHELSA (version 1.2). IPSL-CM6A-LR was developed at the Institute Pierre-Simon Laplace (IPSL) to study natural climate variability and climate response to natural and anthropogenic forces as part of the sixth phase of the Coupled Model Intercomparison Project (CMIP6) [23]. Here, the latest “Shared Socioeconomic Pathways (SSPs)” scenarios of projected socioeconomic global changes up to 2100 from the CMIP6 were used to model the distributions of the Iranian freshwater fish protozoa in the future under the changing global environment [24]. These updated scenarios are called SSP1-2.6, SSP2-4.5, SSP3-7.0, and SSP5-8.5, and the numerical values of the representative concentration pathways (RCPs; 2.6, 4.5, 7.0, and 8.5) refer to the possible range of radiative forcing values in the year 2100. The RCP2.6 is regarded as “the minimum greenhouse gas emission scenario”, while RCP 4.5 and RCP 7.0 reveal the “medium-to-high end of the range of future emissions and warming”. RCP 8.5 presents a “massive enhancement in greenhouse gas emissions up to the end of the twenty-first century” and is indicated as a high emission [25]. In the present study, three RCPs scenarios, 2.6, 7.0, and 8.5, were considered for the future timeline.

#### 2.5.2. Species Distribution Modeling (SDM) and Statistical Analysis

All environmental and topographic layers were clipped to Iran’s boundaries using the mask tool, then converted to the ASCII format required for distribution modeling. The MaxEnt algorithm in the R environment [26] was used to model the distribution of Iranian freshwater fish protozoa and predict their current and future distributions under climate change [27]. MaxEnt has been shown to perform better than other modeling algorithms. It only requires present occurrence data, producing robust models when the sample size is small [28]. MaxEnt was run with maximum iterations of 1000, a convergence threshold of 0.0001, and 1000 background points. Ten replicates were established for each training partition. A bias file was included to reduce sampling bias by correcting how background values and ensure unique occurrence localities are selected (Phillips et al., 2006 [29]). The area under the receiver operating characteristic curve (AUC) was also calculated to measure model performance. AUC represents the degree of separability and indicates how much the model can distinguish between classes (Phillips et al., 2006 [29]). AUC values vary from 0 to 1; 0.5 and shows model performance not (randomly) fit the data, while <0.5 indicates worse than random; 0.5–0.7 presents poor performance; 0.7–0.9 indicates reasonable or moderate performance; and 0.9 indicates high performance; 0.7–0.9 indicates reasonable or moderate performance; and 0.9 indicates high performance [30]. Contributions of each variable to the habitat model of protozoa were calculated using the software’s built-in jackknife test. The jackknife test (systematically leaving out each variable) was used to measure the dominant climatic factors determining the potential distribution of the species [29].

**Figure 2:**
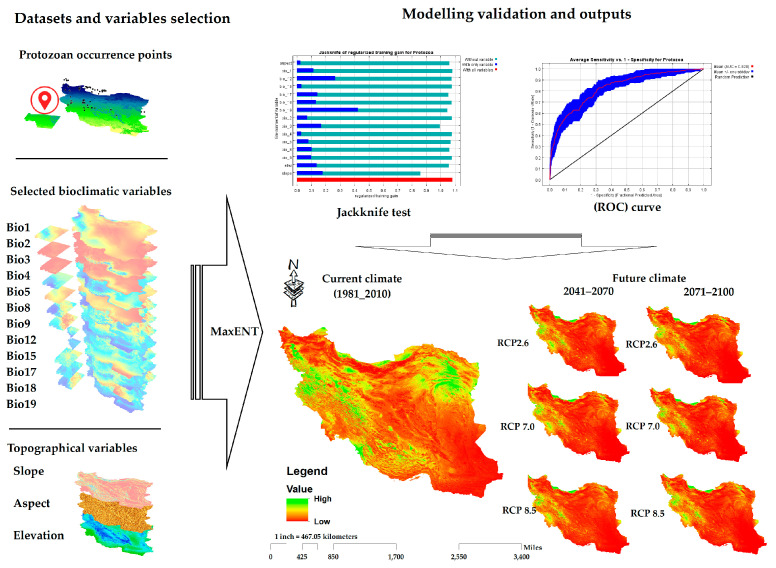
Flowchart of methodological and analytical processes of MaxEnt modelling to project potential distribution of future climatically suitable habitats for freshwater fish protozoa under different global change scenarios.

Finally, the projecting module in the MaxEnt model was used to project the trained models to future scenarios, with 12 changing environmental factors and three unchanged factors. The flow chart of the database and modeling distribution is presented in Figure 2.

## 3. Results

### 3.1. Analysis of Published Reports

Data were obtained from 58 publications (48 scientific articles, two abstracts of conference proceedings, three scientific reports, four student theses, and one book) published between 1981 and 2022. A total of 26 parasite species were recorded from 52 freshwater fish species across different ecoregions of Iran. The protozoan parasites recorded were Tubulinea (1 species), Choanozoa (1 species), Apicomplexa (3 species), Euglenozoa (5 species), Metamonada (1 species), and Ciliophora (15 species). The most common microhabitats in fish were external organs, such as gills, the surface of skin and fins, and the surface of eyes. Gills (filaments, operculum, and gill cavity) were the most commonly infected site, harboring about 15 protozoan parasite species (Table 3).

**Figure 3:**
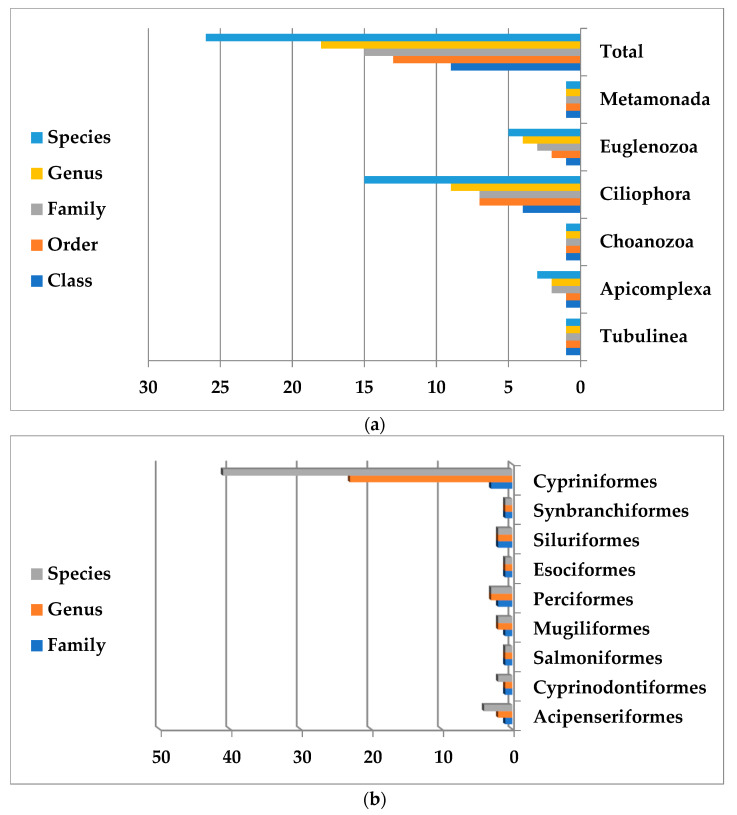
(**a**): Phyla of protozoan parasites found in Iranian freshwater fishes; (**b**): Order of Iranian freshwater fish infected with protozoan parasites.

The broadest host range was observed in holotrichous ciliate, *I. multifiliis*, which infected a broad spectrum of wild and cultured fish species, mostly belonging to the Cyprinidae family. This parasite was isolated from 51 cyprinid fish species belonging to 35 genera from 57 different localities. The highest diversity of protozoan parasites was found in Cypriniform fishes, the most abundant fish species (with 41 members, 72%). The maximum number of parasites, both in terms of the species and abundance, occurred in the native cyprinid fish, *Capoeta capoeta* as follows: *Cyprinus carpio* (Güldenstädt, 1773) (five species), *Ctenopharyngodon idella* (Valenciennes, 1844) (two species), *Hypophthalmichthys molitrix* (Valenciennes, 1844) (four species), *H. nobilis* (Richardson, 1845) (one species), and *Oncorhynchus mykiss* (Walbaum, 1792) (seven species). In addition, protozoan species were reported from wild and cultured sturgeon fish, with at least four identified species in *Acipenser persicus* (Table 4). The Phyla of protozoan parasites found in Iranian freshwater fishes and Orders of Iranian freshwater fish infected with protozoan parasites are presented in Figure 3.

### 3.2. Spatial Analysis

#### Protozoan Occurrence Map

**Figure 4:**
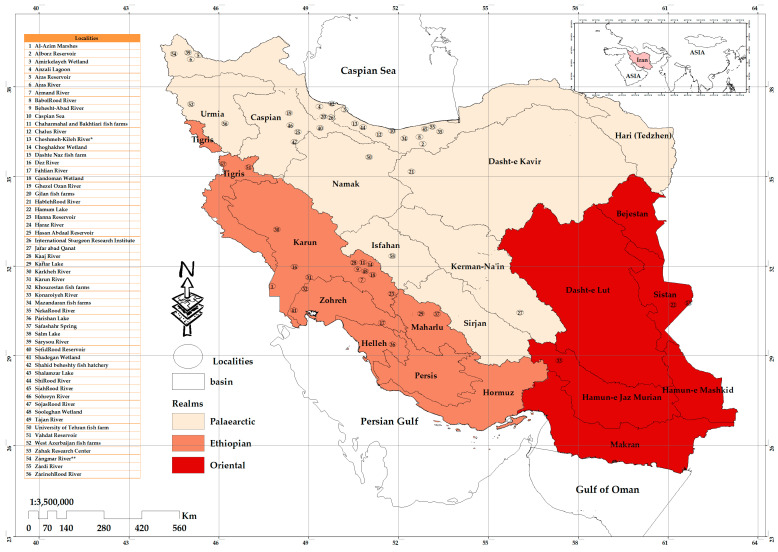
Map of Iran showing main drainage basins and spatial distribution of localities where protozoan species of freshwater fishes have been reported. (* Tonekabon River is considered the Cheshme Kilah River. ** Zangmar River is also known as the Zangbar River).

**Figure 5:**
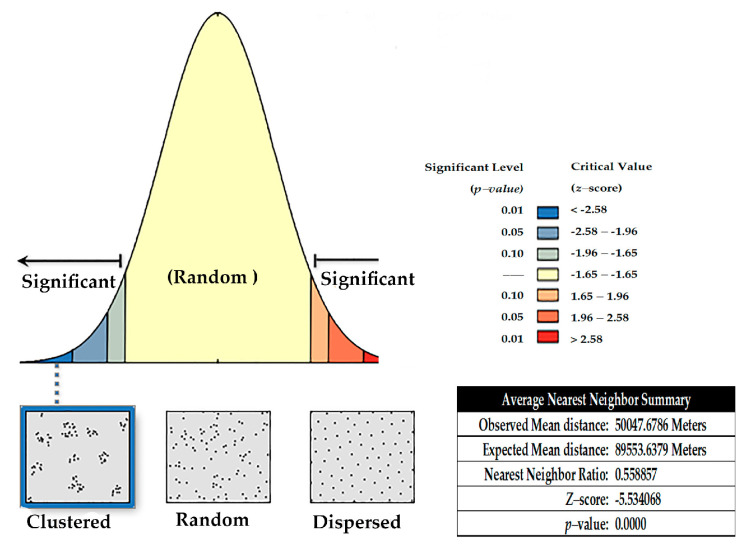
The results for Average Nearest Neighbor.

Drainage basins and spatial distribution of the natural and artificial features for localities where protozoan species of freshwater fishes have been reported are presented in Figure 4. The outcomes from measuring the distance between the center of each feature and its nearest neighbor’s center indicate that the averages of all nearest neighbor distances are less than the average for a hypothetical random distribution (*z*-score = −5.534068; *p*-value < 0.000). This *z*-score indicates that the likelihood of this clustered pattern being random chance is less than 1% (i.e., the distribution of the studied localities was mainly found in clusters within the northern and northwest to southwest parts of the country). The results for average nearest neighbor are presented in Figure 5. 

**Figure 6:**
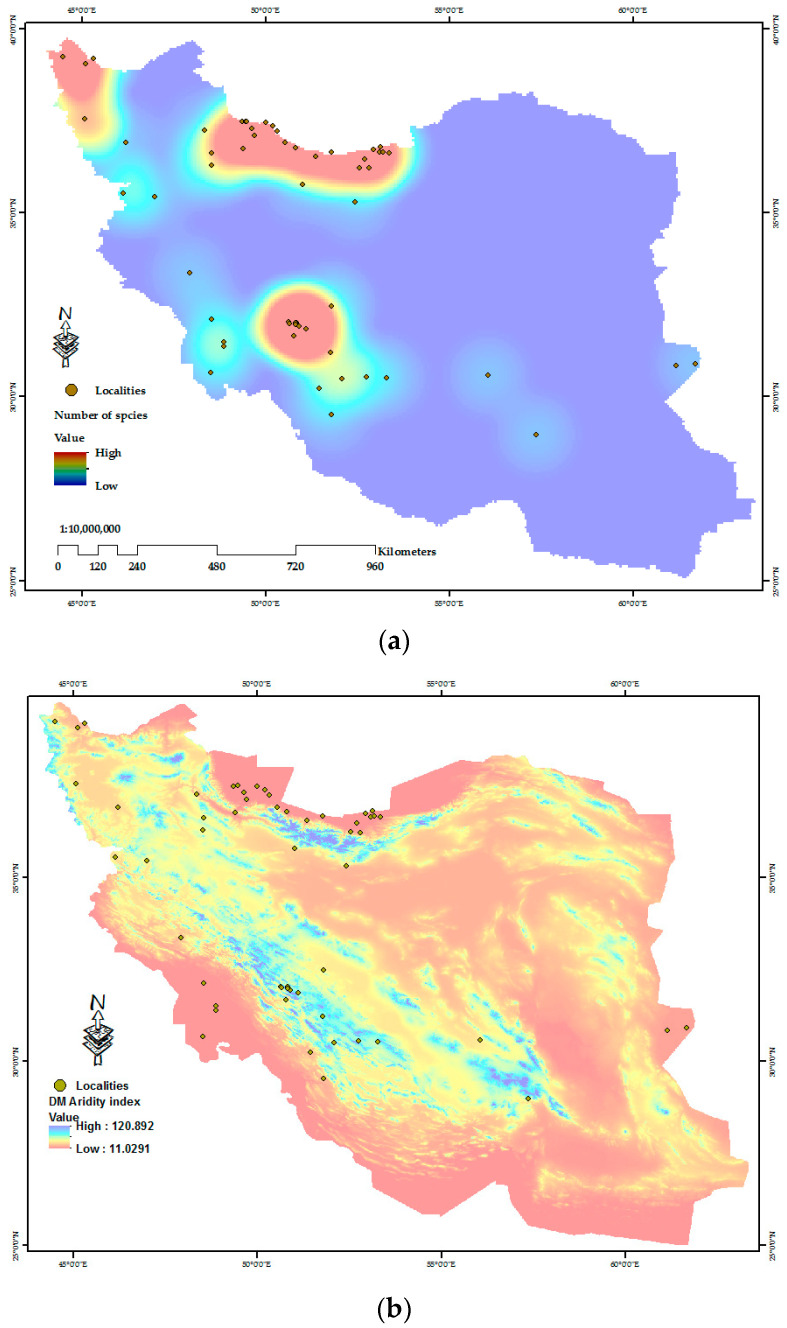
Hotspot mapping of individual protozoan species (**a**); and the protozoan occurrence points relative to the DM aridity index (DMAI) map of Iran, based on the past two years’ average (2020–2022) (**b**). In the top figure red clusters illustrate the higher number of protozoan species, and the lighter-toned cluster zones (light blue) indicate a lower number of species. In the bottom figure red-colored cluster zones indicate a higher DMAI value, while the lighter-toned cluster zones show a lower DMAI value.

The results of kernel density estimation (KDE) modeling to determine important hotspots for protozoan species throughout Iran are shown in Figure 6. The greatest number of protozoan species was reported from the north, northwest, and southwest of the country. Furthermore, presenting the protozoan occurrence points to the spatial distribution map of the DM aridity index indicates that most studies on protozoan parasites have been documented in areas with very humid to Mediterranean climates (Figure 6).

### 3.3. Distribution Modeling

#### 3.3.1. Model Performance and Contribution of Environmental Variables

**Figure 7:**
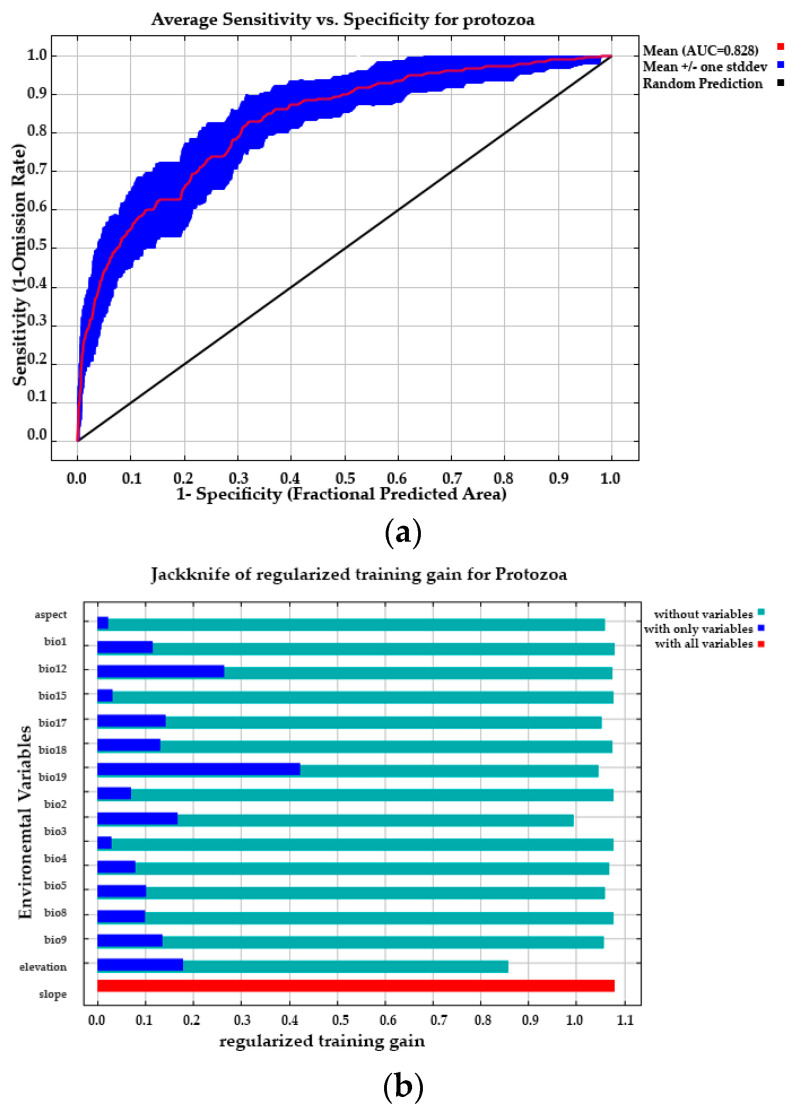
The relative importance of different predictor variables based on the results of jackknife tests in MaxEnt. Graphs represent the contribution of the variables in regularized training test gain (**a**); test gain (**b**).

The accuracy of predicting the probable distribution of freshwater fish protozoa during the current period was found to be “good” (AUC mean = 0.828, which indicates reasonable or moderate performance). The results show that the selected variables described the current distribution of protozoan parasites very well. Among the fifteen environmental and topographical variables, the contribution of four variables, precipitation of coldest quarter (33.9%), slope (22.9%), isothermality (13.9%), and mean temperature of wettest quarter, accounted for 78.6% of model prediction. The results of the jackknife test also show that annual precipitation, annual mean temperature, aspect, and slope were the main variables (Figure 7).

Table 5 represents the mean AUC values of protozoan parasites in the future (2041–2070 and 2071–2100), indicating “good” performance. These findings indicate that the simulations have high reliability and can be used to analyze the impact of climate change on the distribution of freshwater fish protozoan parasites in Iran.

#### 3.3.2. Predicted Current Potential Distribution

**Figure 8:**
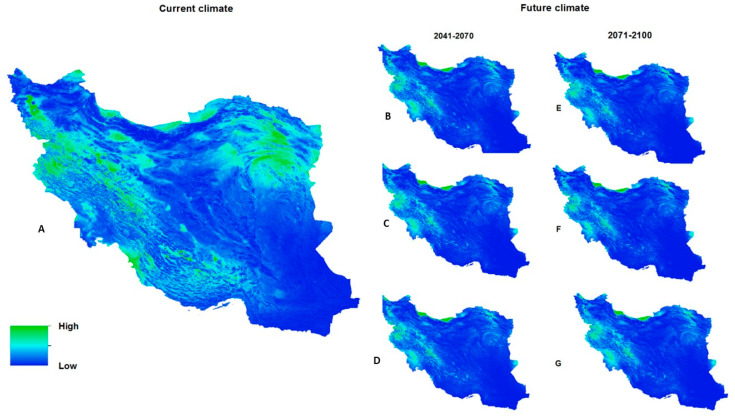
Potential distribution of protozoan parasites of freshwater fishes in Iran; current (**A**) and future distribution; the 2050s (2041–2070) from IPSL-CM6A-LR according to the different climate scenarios (RCPs); RCP2.6 (**B**); RCP 7.0 (**C**); and RCP 8.5 (**D**); and the 2080s (2071–2100) from IPSL-CM6A-LR according to the different climate scenarios (RCPs); RCP2.6 (**E**); RCP 7.0 (**F**); and RCP 8.5 (**G**). Colors display the habitat suitability for fish hosts (green = high suitability).

The distribution map of protozoan parasites of Iranian freshwater fish based on occurrence points, current environmental conditions, and topographic parameters, projected by the MaxEnt model, is presented in Figure 8. The map illustrates that the total suitable habitats, including poorly, moderately, and highly suitable, are widespread throughout the north and west of Iran. However, the northeast of the country might also be a suitable habitat.

#### 3.3.3. Future Suitable Climate Spaces

The potential distribution of future climatically suitable habitats for freshwater fish protozoa under RCP 2.6, RCP 7.0, and RCP 8.5 climate change scenarios for 2041–2070 and 2071–2100 were projected using the MaxEnt model (Figure 8). The findings demonstrate a significant difference between current and predicted total suitable habitats in 2041–2070 (RCP 2.6, RCP 7.0, and RCP 8.5) and 2071–2100 (RCP 2.6, RCP 7.0, and RCP 8.5); in particular, the area size of suitable habitats varies from “keeps up” to “remarkably decreases”.

## 4. Discussion

According to the literature, 24 protozoan species were found in Iranian freshwater fish, and the most commonly reported microhabitats of fish hosts were external organs such as gills, skin and fins, and eyes’ surface. The skin surface and gills (filaments, operculum, and gill cavity) were the most commonly infected sites, harboring 14 and 11 protozoan parasite species, respectively. In contrast, the published data on protozoan infections in the internal organs is limited and mainly focuses on farmed fish species, likely due to global public health concerns. Only eight protozoan species have been reported from the blood (*Trypanoplasma acipenseris*, *T. Borelli*, *Trypanosoma percae*, *Haemogregarina acipenseris*) and gastrointestinal tract (*Hexamita salmonis*, *Balantidium ctenopharyngodoni*, *Eimeria carpelli*, *Eimeria sinensis*) of sturgeon and cultured carp [7,10,82]. Notably, the examined fish are usually dead when obtained from the market or sent to the laboratory, making internal and blood parasites difficult to study and potentially confounding the reported data.

In the current study, a slight decrease in the number of identified protozoan species was observed compared to the checklist by Pazooki and Masoumian [10]. The only new record is *Trichodina gracilis*, which was isolated on the gills of *Capoeta razii* from BabolRood River [62]. However, some taxonomic groups have been changed, and some reported species are no longer classified as parasites. Genus *Pleistophora* (Gurley, 1893) belongs to the Microsporidia phylum, which has traditionally been considered protozoan, but is now classified within the kingdom Fungi according to recent molecular phylogeny [83]. Furthermore, some questionable taxonomies, e.g., *Cryptobia linchi*, listed by Pazooki and Masoumian [10], seem to be misspelled in recorded data, and some modifications have been made to the taxonomic validity of *Cryptobia acipenseris* and *C*. *Borelli* [10]. Lom and Dykova [84] stated that *Cryptobia* and *Trypanoplasma* are morphologically similar, but based on their host infection site, the ectocommensal group is considered a species of *Cryptobia* and another living in the bloodstream as *Trypanoplasma*. Transmission of genus *Cryptobia* is direct (host to host) without any developmental changes, while the latter are transmitted by hematophagous leeches in which some development stages of the parasite occur [15]. In the present checklist, these two protozoan parasites are under the genus *Trypanoplasma* (Laveran and Mesnil, 1901). Protozoan ciliate *Trichodina epizootica*, documented by Rahanandeh and Tizkar [67] from the skin and fins of *H. molitrix* is now classified under the genus *Trichodinella* (Srámek-Husek, 1953) in the World Registry of Marine Species. As the morphological or molecular characteristics of the parasite were not cited in their research work, the parasite is excluded from the present list.

There is more information available on protozoan parasites, namely *Trichodina*, *Ichthyobodo*, and *Chilodonella*, than other protozoa [11,12,54,63]. Since there may be new species, and/or information about different localities and host species, further collaboration among researchers in various fields of parasitology is essential. Moreover, the development of the best methods for collecting and preserving protozoan parasite specimens, and applying novel laboratory diagnostics (e.g., molecular procedures) is pivotal to the accurate parasite description and identification.

The most prevalent species was *I. multifiliis*, which was reported from 57 different water resources in the country. The main host for this parasite is the common carp, which is widely cultivated in farms and natural water resources throughout Iran; thus, the wide distribution of the parasite may have occurred during the introduction of common carp and other Chinese carp [7].

Specific identification of protozoa can be challenging. Molecular taxonomy has changed the taxonomic status and phylogenetic relationship of many protozoan taxa. For example, myxosporidians are indeed no longer classified as protozoa and are instead considered metazoan organisms. They have been included in this study based on their previous classification. Unfortunately, molecular taxonomy of parasites in Iran lags behind the rest of the world. In particular, there are no sequence data for protozoan parasites of Iranian freshwater fish.

### 4.1. Host-Parasite List

The highest diversity in protozoan parasites belonged to Cyprinidae, with the maximum number of individual parasite species in *Capoeta capoeta*. *Trichodina gracilis* recorded for the first time from the gills of *Capoeta razii* by Mirnategh, Shabanipour, and Sattari [77]. *C. razii* was first described by Jouladeh-Roudbar et al. [85] from the KheyRood River, in the southern part of the Caspian Sea basin, as an endemic species. They stated that the genus *Capoeta* in the southern Caspian Sea Basin comprised two species, namely *C. capoeta* and the new species, *C. razii,* which differ molecularly and morphologically from other described *Capoeta* species. As the highest number of reported protozoan species belonged to the genus Capoeta, there may be more individual species in *C. razii* that need further investigation.

Among the reported parasites, the widest host range was observed in the ciliated ectoparasitic protozoan, *I. multifiliis*. The parasite causes ichthyophthiriasis or white spot disease and is one of the most economically important freshwater parasites globally [86]. *I. multifiliis* has a broad host range and was isolated from the skin, fins, and surface of the eyes of a broad spectrum of wild and cultured fish species from the Cyprinidae family (51 species belonging to 35 various genera).

Currently, the number of freshwater fish species in Iran is 297 [81], of which 57 fish species have been reported to be infected with parasites, accounting for only 19.2% of Iran’s fish diversity. Cyprinids, sturgeons, and salmonids have been evaluated for parasites more frequently and in more localities. However, most Iranian fishes have been examined for parasites only on a single occasion or not at all. This could be attributed to these species being rare, with some being very difficult or expensive to access. Moreover, some species are not considered important enough for parasitological examination.

In terms of host specificity, clearly some parasites, such as *I. multifiliis*, infect a broad range of hosts in different families, environments, and host age groups. Others such as *Trichodina* spp. can be fatal to juvenile fish but not adults, and some, such as *Chilodenella* spp., can be free living and become parasitic when the environment changes or the fish is under stress.

### 4.2. Mapping Distribution

In the environment, each parasite species occupies a particular niche. In addition to their microhabitats (infected organs), parasites are found in macrohabitats, which are part of the host habitat. However, macrohabitats and geographical ranges cannot always be clearly differentiated [87]. The geographical distribution of a parasite can be influenced by various host- and environment-dependent factors [88]. Behavioral and physiological characteristics of hosts (e.g., diet, migratory behavior, and defecation) can determine the parasite type/s encountered by the host [89], while environmental conditions can facilitate parasite viability and establishment [90]. Thus, our spatial distribution map of localities where infected fish species were caught also shows the parasite macrohabitats/geographical ranges and forms the basis for modeling current and future parasite distributions under different global change scenarios. Our results show that protozoan parasite distributions primarily occur in clusters in northern and northwest to southwest Iran. This indicates that the total suitable macrohabitats are mainly in the Palaearctic and Ethiopian Realms, which are both considered ecologically important, having substantial water resources and numerous diverse freshwater fish species [91]. Overlaying the occurrence points on the spatial distribution map of the DM aridity index indicated that most of the literature on protozoan parasites has been documented in very humid to Mediterranean climate types. The outcome of KDE for hotspot mapping confirmed this finding. Accordingly, the greatest number of protozoan species was reported from the north, northwest, and southwest, indicating the extent and abundance of suitable aquatic macrohabitats for protozoan parasites in these areas. It is noted that these areas may be more intensively studied due to accessibility to fish hosts, and proximity to laboratories and research centers.

Our potential distribution map of protozoan parasites of Iranian freshwater fish based on occurrence points, current environmental conditions, and topographic parameters was projected using the MaxEnt model for current and future scenarios. The results showed reasonable or moderate performance, which means that the potential distribution map created using MaxEnt is reliable. Similarly, most of the available literature emphasized that the maximum entropy (Maxent) could be a powerful predictive technique for ecological niche modeling of aquatic species, particularly fish species and their specific parasites [92,93,94]. The outcomes of the jackknife test indicated that precipitation and temperature played the most critical roles in predicting the probable distribution of freshwater fish protozoa throughout Iran. Similarly, Yousefi et al. [95] modeled the potential distribution of 15 endemic freshwater species under climate change in Iran and suggested that precipitation was the most crucial determinant of fish distribution, while Kim et al. [96] showed that temperature had the highest contribution to largemouth bass (*Micropterus salmoides*) distribution in South Korea.

The outcomes of the current study in relation to the potential distribution map for the current period demonstrated that the total suitable habitats for protozoan parasites are basically widespread throughout the north and west of the country. However, the northeast of the country may also be a suitable ecological niche. There is no research on freshwater fish parasites in this area despite providing natural and artificial habitats for many fish species [91]. Furthermore, a remarkable difference was observed in the model comparison of current and future protozoan parasites’ potential distribution places. This suggests that as fish host habitats shrink, protozoan parasite species also lose suitable habitat and geographical range.

There is very little research on modeling the distribution and predicting environmentally suitable habitats of freshwater fish parasites, and most of the available studies deal with marine species [93,97]. However, our findings for habitat reduction align with previous research that predicted range reductions for different groups of freshwater fish. Esmaeili, Sayyadzadeh, Eagderi, and Abbasi [81] showed that climate change might negatively affect the distribution of *Alburnus* species in Iran. They asserted that the current potential suitable places for this species would decrease in future. Similarly, Kwon et al. [98] projected the current and future distribution of some endemic freshwater fish in Korea under the RCP 8.5 scenario and revealed that climate change would probably lead to a decrease in the range size of suitable predicted spaces for some fish species. Pandit et al. [99] predicted the potential distribution of the threatened freshwater fish, Carmine shiner (*Notropis percobromus*), under various climate change scenarios, concluding that the available predicted areas for Carmine shiner would significantly decrease.

Iranian natural freshwater ecosystems are mainly identified as endorheic basins—land-locked drainage networks with no hydrological connection with marine environments [91]. Natural topographic barriers, basin fragmentation due to climate change and the resulting drought [100], and anthropogenic interventions may negatively affect occupants of aquatic systems and their interactions. These provide barriers that impede intracontinental migration in an endorheic system. Consequently, it would be difficult for fish species and parasites to change their distribution ranges to more suitable climates. Accordingly, it can be anticipated that climate change may lead to a shift in latitudinal and elevational distribution ranges [98,101], population decline in some species, or co-extinction of the host-specific parasites. In farmed fish, protozoan parasites are affected by health management policies and environmental changes. Unauthorized transport of live or harvested fish, substandard health conditions in some farms, intensive culture, and lack of disinfection have resulted in parasite establishment and geographical dispersal [101].

Currently, some parts of Iran, including the eastern regions of the Sistan Basin, Hamoon Lake, the southern regions of the Karun Basin, and the Iranian part of Al-Azim Marshes [102] are suffering from severe drought, primarily due to climate change. Future climate change is predicted to further increase temperatures and decrease precipitation, intensifying drought severity [103]. This, in turn, will threaten freshwater ecosystems, making them less or more habitable for fish species and their parasites. Moreover, habitat destruction caused by oil and gas projects, wastewater discharge, dam construction, and land-use changes can accelerate the adverse effects of climate modification [104,105], which should be considered in future research.

## 5. Conclusions

This paper has provided updated information regarding protozoan parasites of freshwater fish in Iran and a host–parasite list that may be utilized in future studies. Approximately one-sixth of freshwater fish reported in Iran were infected with protozoa, and most of the parasitic diversity found was related to the Cyprinidae family. Due to the great richness of freshwater fish species and extreme diversity in habitats, parts of the parasite fauna of Iranian freshwater fish are possibly poorly known. Protozoan infection has been documented in almost all economically important fish species such as cyprinids, sturgeons, and salmonids. The most prevalent protozoan species was I. multifiliis, which was reported in over two-thirds of the literature and was isolated from a range of wild and cultured fish species from the Cyprinidae family.

Distribution modeling underlined that MaxEnt could accurately predict habitat location and distribution for fish parasites, and mapping of future potential distribution demonstrated that northeastern Iran might also be a suitable ecological niche. In addition, the model comparison of current and future protozoan parasites’ potential distribution revealed that future climate change followed by intensifying droughts could affect parasite populations due to changes in fish hosts and suitable habitats.

## Figures and Tables

**Table 1:** Types of climate according to the de Martonne aridity index (IDM).

Climate Type	IDM Values
Arid	IDM < 10
Semi-arid	10 ≤ IDM < 20
Mediterranean	20 ≤ IDM < 24
Semi-humid	24 ≤ IDM < 28
Humid	28 ≤ IDM < 35
Very humid	35 ≤ IDM < 55

**Table 2:** List of predictor variables selected primarily to simulate the distributions of fish protozoan parasites in Iran.

Categories	Name of Variables	Unit
Bioclimatic variables	Annual mean temperature (BIO1)	°C
	Mean diurnal range (mean of monthly max temp–min temp) (BIO2)	°C
	Isothermality (BIO2/BIO7) (×100) (BIO3)	°C
	Temperature seasonality (standard deviation ×100) (BIO4)	°C
	Max temperature of warmest month (BIO5)	°C
	Mean temperature of wettest quarter (BIO8)	°C
	Mean temperature of driest quarter (BIO9)	°C
	Annual precipitation (BIO12)	mm
	Precipitation seasonality (coefficient of variation) (BIO15)	mm
	Precipitation of driest quarter (BIO17)	mm
	Precipitation of warmest quarter (BIO18)	mm
	Precipitation of coldest quarter (BIO19)	mm
Topography variables	Elevation (DEM)	m
	Slope	%
	Aspect (Asp)	Degrees

**Table 3:** Details of protozoan parasites reported in Iranian freshwater fish. Data are sorted alphabetically based on parasite taxonomy, followed by host taxonomy. The water basin type in which the parasite was reported is provided as FW for freshwater and B for brackish water.

Parasite	Host Taxonomy	Host Name	Infected Organ	Environment, Type	Basin	Locality	Ref.
Ph: Apicomplexa Cl: Conoidasida Or: Eucoccidiorida Fa: Eimeriidae							
***Goussia carpelli*** **(Leger and Stankovich, 1921) Dykova and Lom, 1983**	OR: Cypriniformes FA: Cyprinidae	*Cyprinus carpio*	Intestine	FW, Re, Ri	Caspian	Aras, SefidRood	[31,32]
***Goussia sinensis*** **Chen, 1956**	OR: Cypriniformes FA: Xenocyprididae	*Hypophthalmichthys* *molitrix*	Intestine	FW, Ri	Caspian	SefidRood	[32]
**Ph: Apicomplexa** **Cl: Conoidasida** **Or: Eucoccidiorida** **Fa: Haemogregarinidae**							
***Haemogregarina acipenseris* ** **Navrotskii, 1914**	OR: Acipenseriformes FA: Acipenseridae	*Acipenser* *gueldenstaedtii*	Blood	BW, S	Caspian	Caspian	[33]
		*Acipenser* *persicus*	Blood	BW, S	Caspian	Caspian	[33]
**Ph: Choanozoa** **Cl: Ichthyosporea** **Or: Dermocystida** **Fa: Dermocystidae**							
***Dermocystidium salmonis*** **Davis 1947**	OR: Salmoniformes FA: Salmonidae	*Oncorhynchus mykiss*	Gills	FW, Fa	Tigris	Chaharmahal and Bakhtiari	[10]
**Ph: Ciliophora** **Cl: Litostomatea** **Or: Pleurostomatida** **Fa: Amphileptidae**							
***Amphileptus branchiarum*** **Weinrich, 1924**	OR: Cypriniformes FA: Cyprinidae	*Capoeta capoeta*	Skin, fins	FW, Ri	Urmia	Zangmar	[8]
**Ph: Ciliophora** **Cl: Litostomatea** **Or: Vestibuliferida** **Fa: Balantidiidae**							
***Balantidium ctenopharyngodoni*** **Chen, 1955**	OR: Cypriniformes FA: Xenocyprididae	*Ctenopharyngodon idella*	Intestine	FW, L	Sistan	Hamun	[34]
**Ph: Ciliophora** **Cl: Oligohymenophorea** **Or: Hymenostomatida** **Fa: Ichthyophthiriidae**							
***Ichthyophthirius multifiliis*** **Fouquet, 1876**	OR: Acipenseriformes FA: Acipenseridae	*Acipenser* *persicus*	Gills	FW, Fa	Caspian	International Sturgeon Fa	[35]
	OR: Acipenseriformes FA: Acipenseridae	*Acipenser* *stellatus*	Gills	FW, Fa	Caspian	International Sturgeon Fa	[35]
	OR: Acipenseriformes FA: Acipenseridae	*Huso huso*	Gills	FW, Fa	Caspian	International Sturgeon Fa	[36]
	OR: Cypriniformes FA: Cyprinidae	*Arabibarbus grypus*	Skin, gills	FW, Ri, W	Karun; Tigris	Karun, Karkheh, Shadegan; Armand	[37,38]
		*Barbus lacerta*	gills	FW, Ri	Kavir	HablehRood	[39]
		*Capoeta aculeata*	Gills, skin	FW, Ri, W	Isfahan; Tigris; Kavir	ZayandehRood; Armand, Choghakhor; Kaaj; HablehRood	[38,39,40,41,42]
		*Capoeta barroisi*	Gills, skin	FW, Ri	Kor	Fahlian	[43]
		*Capoeta capoeta*	Gills, skin	FW, Ri	Caspian; Isfahan; Urmia	SefidRood, NekaRood, SojasRood; ZayandehRood; Sarysou, Zangmar	[8,9,44,45,46]
		*Capoeta damascina*	Gills, skin	FW, Q, Ri, W	Kerman- Nain; Isfahan; Tigris, Urmia; Kavir	Jafar abad, Konaroiyeh; ZayandehRood; Armand, Kaaj; Choghakhor; ZarinehRood; HablehRood	[7,38,39,40,41,42,44,47]
		*Capoeta trutta*	Gills, skin	FW, Ri	Karun	Dez	[48]
		*Carasobarbus luteus*	Gills, skin	FW, L, W	Kor; Karun	Parishan; HoorAlazim	[49,50]
		*Carassius carassius*	Gills, skin	FW, L, Re	Kor; Urmia	Parishan; Aras	[8,49]
		*Carassius auratus*	Gills, skin	FW, Ri, W	Tigris; Caspian; Isfahan	Choghakhor; SefidRood; Hanna	[9,41,51]
		*Carassius gibelio*	Gills, skin	FW, Ri, Lg, W	Urmia; Caspian; Tigris	ZarrinehRood; Anzali, SefidRood; Gandoman,	[9,52,53,54,55]
						Sooleghan	
		*Cyprinion macrostomum*	Gills, skin	FW, Ri	Karun; Kor	Dez; Fahlian	[43,48]
		*Cyprinus carpio*	Gills, skin, fin	FW, Ri, Re, Fa, Lg, W, L	Urmia; Caspian; Karun; Tigris; Kor; Isfahan	ZarrinehRood; Mazandaran, Anzali, Dashte Naz, SefidRood; HoorAlazim, Sooleghan, Vahdat, Gandoman; Parishan; ZayandehRood	[9,40,49,50,52,54,55,56,57,58,59]
		*Luciobarbus barbulus*	Gills, skin	FW, Ri	Karun; Tigris	Dez; Armand	[38,48]
		*Luciobarbus capito*	Skin	FW, Ri	Caspian	Aras	[8]
		*Luciobarbus* *esocinus*	Skin	FW, Ri, W	Karun	Karun, Karkheh, Shadegan	[37]
		*Mesopotamichthys sharpeyi*	Gills, skin	FW, Ri, W	Karun	Karun, Karkheh, Shadegan, HoorAlazim	[37,50]
		*Schizocypris* *altidorsalis*	Skin	FW, L	Sistan	Hamun	[34]
		*Schizothorax pelzami*	Skin	FW, L	Sistan	Hamun	[34]
		*Schizothorax* *zarudnyi*	Gills, skin	FW, L, Fa	Sistan	Hamun, Zahak	[34,60]
	OR: Cypriniformes FA: Leuciscidae	*Abramis brama*	Skin	FW, Re	Urmia	Aras	[8]
		*Acanthobrama persidis*	Gills	FW, L	Kor	Kuftar	[61]
		*Alburnoides eichwaldii*	Skin	FW, Ri	Caspian	Aras	[8]
		*Alburnoides tabarestanensis*	Gills	FW, Re, Ri	Caspian	Alborz, BabolRood	[62]
		*Alburnus* *chalcoides*	Gills	FW, Ri	Isfahan; Caspian	ZayandehRood; Cheshmeh Kileh, ShiRood	[6,40]
		*Alburnus* *hohenackeri*	Gills, skin	FW, L	Tigris	Zarivar	[63]
		*Alburnus* *mossulensis*	Gills, skin	FW, Ri	Kor	Fahlian	[43]
		*Chondrostoma orientale*	Gills, skin	FW, L, W	Kor; Tigris	Kuftar; Choghakhor	[41,61]
		*Chondrostoma regium*	Gills, skin	FW, Ri	Tigris; Isfahan	Kaaj, Behesht Abad; ZayandehRood	[42,44,64]
		*Leuciscus vorax*	Gills, skin	FW, W	Karun	HoorAlazim	[50]
		*Squalius* *cephalus*	Gills	FW, Ri	Urmia; Caspian	ZarinehRood;	[7]
						NekaRood, Chalus, Tajan, ShiRood, SiahRood	[46,65,66]
		*Vimba vimba*	Gills	FW, Ri	Caspian	Cheshmeh Kileh	[65]
	OR: Cypriniformes FA: Xenocyprididae	*Ctenopharyngodon idella*	Gills	FW, Ri	Isfahan; Caspian; Urmia	ZayandehRood; SefidRood; ZarrinehRood	[9,40,52]
		*Hemiculter* *leucisculus*	not stated	FW, Lg	Caspian	Anzali	[56]
		*Hypophthalmichthys* *molitrix*	Gills, skin, fin	FW, Fa, Ri	Caspian	Gilan, SefidRood, Mazandaran	[9,57,67]
		*Hypophthalmichthys nobilis*	Skin, fin	FW, Fa	Caspian	Mazandaran	[57]
	OR: Cyprinodontiformes FA: Aphaniidae	*Aphanius* *sophiae*	Gills, skin	FW, Sp	Kor	Safashahr	[68]
		*Aphanius* *vladykovi*	Gills, skin	FW, Ri, L	Isfahan; Tigris	ZayandehRood; Behesht Abad, Shalamzar, Salm	[40,64,69]
	OR: Esociformes FA: Esocidae	*Esox lucius*	Gills	FW, Lg, Ri	Caspian	Anzali, ShiRood	[56,65,70]
	OR: Mugiliformes FA: Mugilidae	*Chelon auratus*	Gills, skin	FW, Ri	Caspian	Zardi	[11]
		*Planiliza abu*	Gills, skin	FW, W	Karun	HoorAlazim	[50]
	OR: Perciformes FA: Gasterosteidae	*Gasterosteus aculeatus*	Gills, skin	FW, Ri	Caspian	Zardi	[11]
	OR: Salmoniformes FA: Salmonidae	*Oncorhynchus mykiss*	Surface of eyes, gills, skin, fin	FW, Ri, Fa	Caspian	Haraz, Mazandaran, Chalus	[10,57,71,72,73]
	OR: Siluriformes FA: Sisoridae	*Glyptothorax silviae*	Gills, skin	FW, Ri	Tigris	Armand	[38]
		*Silurus glanis*	Gills, skin	FW, Lg, Re	Caspian; Urmia	Anzali; Aras	[60,74]
	OR: Synbranchiformes FA: Mastacembelidae	*Mastacembelus mastacembelus*	Gills	FW, L	Tigris	Zarivar	[63]
**Ph: Ciliophora** **Cl: Oligohymenophorea** **Or: Hymenostomatida** **Fa: Tetrahymenidae**							
***Tetrahymena pyriformis*** **Ehrenberg, 1830**	OR: Cypriniformes FA: Cyprinidae	*Capoeta capoeta*	Skin	FW, Ri	Urmia	Zangmar	[8]
**Ph: Ciliophora** **Cl: Oligohymenophorea** **Or: Mobilida** **Fa: Trichodinidae**							
***Trichodina domerguei*** **Wallengren, 1897**	OR: Cypriniformes FA: Cyprinidae	*Barbus lacerta*	Gills, skin	FW, Ri	Caspian	SefidRood	[9]
		*Capoeta capoeta*	Gills, skin	FW, Ri	Caspian	SefidRood	[9]
		*Cyprinus carpio*	Gills, skin	FW, Ri	Caspian	SefidRood	[9]
		*Luciobarbus capito*	Gills, skin	FW, Ri	Caspian	SefidRood	[9]
	OR: Cypriniformes FA: Leuciscidae	*Abramis brama*	Gills, skin	FW, Ri	Caspian	SefidRood	[9]
		*Alburnoides eichwaldii*	Gills, skin	FW, Ri	Caspian	SefidRood	[9]
***Trichodina gracilis*** **Polyanski, 1995**	OR: Cypriniformes FA: Cyprinidae	*Capoeta razii*	Gills	FW, Ri	Caspian	BabolRood	[62]
***Trichodina perforata*** **Lom, Golemansky and Grupcheva, 1976**	OR: Cypriniformes FA: Cyprinidae	*Barbus lacerta*	Gills, skin	FW, Ri	Urmia	SojasRood	[45]
		*Capoeta capoeta*	Gills, skin	FW, Ri, Re	Urmia	Zangmar, Ghezel Ozan, SojasRood, Hasan Abdaal	[8,45]
		*Carassius* *auratus*	Gills, skin, fins	FW, Re	Isfahan	Hanna	[51]
		*Luciobarbus capito*	Gills, skin	FW, Ri	Urmia	Aras, Sarysou	[8]
	OR: Cypriniformes FA: Leuciscidae	*Abramis brama*	Gills, skin	FW, Re	Urmia	Aras	[8]
		*Alburnoides eichwaldii*	Gills, skin	FW, Ri	Urmia	Aras, Sarysou	[8,45]
		*Alburnus* *filippii*	Skin	FW, Ri	Urmia	SojasRood	[45]
		*Blicca bjoerkna*	Gills, skin, fins	FW, Lg	Caspian	Anzali	[75]
	OR: Cypriniformes FA: Xenocyprididae	*Hemiculter* *leucisculus*	Gills, skin, fins	FW, Lg	Caspian	Anzali	[75]
	OR: Perciformes FA: Percidae	*Sander* *lucioperca*	Gills, skin	FW, Re	Urmia	Aras	[8]
	OR: Siluriformes FA: Siluridae	*Siluris glanis*	Gills, skin	FW, Re	Urmia	Aras	[8]
***Trichodina pediculus*** **Ehrenberg, 1831**	OR: Cypriniformes FA: Cyprinidae	*Capoeta* *damascina*	Gills	FW, L	Tigris	Zarivar	[63]
		*Cyprinus carpio*	Gills	FW, L	Tigris	Zarivar	[63]
	OR: Synbranchiformes FA: Mastacembelidae	*Mastacembelus mastacembelus*	Gills	FW, L	Tigris	Zarivar	[63]
***Trichodina truttae*** **Mueller, 1937**	OR: Salmoniformes FA: Salmonidae	*Oncorhynchus mykiss*	Skin, fins	FW, Ri	Caspian	Chalus	[72]
***Trichodina reticulata*** **Hirschmann and Partsch, 1955**	OR: Acipenseriformes FA: Acipenseridae	*Acipenser* *gueldenstaedtii*	not stated	FW, Fa	Caspian	Shahid Beheshty	[76]
		*Acipenser* *persicus*	not stated	FW, Fa	Caspian	Shahid Beheshty	[76]
		*Acipenser* *stellatus*	not stated	FW, Fa	Caspian	Shahid Beheshty	[76]
	OR: Mugiliformes FA: Mugilidae	*Chelon auratus*	not stated	BW, S	Caspian	Kiashahr, Anzali, Chamkhaleh	[77]
***Trichodinella subtilis*** **Lom, 1959**	OR: Cypriniformes FA: Cyprinidae	*Cyprinus carpio*	Skin	FW, Fa	Urmia	West Azerbaijan	[10]
***Tripartiella lata*** **Lom 1963**	OR: Salmoniformes FA: Salmonidae	*Oncorhynchus mykiss*	Skin	FW, Fa	Urmia	West Azerbaijan	[10]
**Ph: Ciliophora** **Cl: Phyllopharyngea** **Or: Chlamydodontida** **Fa: Chilodonellidae**							
***Chilodonella cyprini*** **(Moroff, 1902) Strand, 1928**	OR: Cyprinodontiformes FA: Aphaniidae	*Squalius* *cephalus*	Skin	FW, Ri	Caspian	Chalus	[72]
	OR: Salmoniformes FA: Salmonidae	*Oncorhynchus mykiss*	Skin	FW, Fa, Ri	Tigris; Caspian	Chaharmahal and Bakhtiari; Chalus	[10,72]
***Chilodonella piscicola*** **(Zacharias 1894) Jankowski 1980**	OR: Cypriniformes FA: Cyprinidae	*Capoeta capoeta*	Gills, skin	FW, Ri	Urmia	Ghezel Ozan	[45]
	OR: Cypriniformes FA: Xenocyprididae	*Hypophthalmichthys* *molitrix*	Gills, skin, fins	FW, Fa	Caspian	Gilan	[67]
**Ph: Ciliophora** **Cl: Spirotrichea** **Or: Sporadotrichida** **Fa: Oxytrichidae**							
***Stylonychia pustulata*** **(Müller, 1786) Ehrenberg, 1835**	OR: Cypriniformes FA: Cyprinidae	*Capoeta capoeta*	Gills	FW, Ri	Urmia	Zangmar	[8]
**Ph: Euglenozoa** **Cl: Kinetoplastea** **Or: Ichthyobodonidae** **Fa: Ichthyobodonidae**							
***Ichthyobodo necator*** **Henneguy, 1928**	OR: Cypriniformes FA: Cyprinidae	*Arabibarbus grypus*	Skin	FW, W	Karun	HoorAlazim	[7]
		*Capoeta capoeta*	Gills	FW, Ri, Re	Urmia; Caspian	ZarinehRood; Sohreyn	[7,45]
		*Carassius auratus*	Skin	FW, Lg	Caspian	Anzali	[10]
	OR: Cypriniformes FA: Leuciscidae	*Leuciscus vorax*	Gills	FW, W	Karun	HoorAlazim	[7]
	OR: Cyprinodontiformes FA: Aphaniidae	*Aphanius* *vladykovi*	Skin	FW, L	Tigris	Shalamzar	[69]
	OR: Cypriniformes FA: Xenocyprididae	*Hemiculter* *leucisculus*	Skin	FW, Ri	Caspian	Zardi	[11]
	OR: Mugiliformes FA: Mugilidae	*Chelon auratus*	Skin	FW, Ri	Caspian	Zardi	[11]
		*Planiliza abu*	Skin, gills	FW, W, Ri	Karun	HoorAlazim, Karun	[7]
**Ph: Euglenozoa** **Cl: Kinetoplastea** **Or: Parabodonida** **Fa: Cryptobiaceae**							
***Cryptobia branchialis*** **Nie in Chen, 1956**	OR: Cypriniformes FA: Xenocyprididae	*Hypophthalmichthys* *molitrix*	Gills, skin, fins	FW, Fa	Karun; Caspian	Khouzestan; Guilan	[7,67]
**Ph: Euglenozoa** **Cl: Kinetoplastea** **Or: Parabodonida** **Fa: Cryptobiaceae**							
***Trypanoplasma acipenseris*** **Ioff, Lewashow, Boschenko, 1926**	OR: Acipenseriformes FA: Acipenseridae	*Acipenser* *gueldenstaedtii*	Blood	BW, S	Caspian	Caspian	[33]
	OR: Acipenseriformes FA: Acipenseridae	*Acipenser persicus*	Blood	BW, S	Caspian	Caspian	[33]
***Trypanoplasma borelli*** **Laveran et Mesnil, 1901**	OR: Salmoniformes FA: Salmonidae	*Oncorhynchus mykiss*	Blood	FW, Fa	Tigris	Chaharmahal and Bakhtiari	[78]
**Ph: Euglenozoa** **Cl: Kinetoplastea** **Or: Trypanosomatida** **Fa: Trypanosomatidae**							
***Trypanosoma percae*** **Brumpt, 1906**	OR: Cypriniformes FA: Leuciscidae	*Alburnus* *chalcoides*	Blood	FW, Ri	Caspian	Sefidrood	[79]
	OR: Perciformes FA: Percidae	*Perca fluviatilis*	Blood	FW, W, S	Caspian	Amirkelayeh, Caspian	[7,80]
**Ph: Fornicata** **Cl: Trepomonadea** **Or: Diplomonadida** **Fa: Hexamitidae**							
***Hexamita salmonis*** **(Moore, 1923) Wenyon, 1926**	OR: Salmoniformes FA: Salmonidae	*Oncorhynchus mykiss*	Intestine	FW, Fa	Tigris; Urmia	Chaharmahal and Bakhtiari; West Azerbaijan	[10,78]
**Ph: Tubulinea** **Cl: Elardia** **Or: Arcellinida** **Fa: Arcellidae**							
***Arcella vulgaris*** **Ehrenberg, 1830**	OR: Cypriniformes FA: Cyprinidae	*Capoeta capoeta*	Skin	FW, Ri	Urmia	Zangmar	[8]

Fa: fish farm, L: lake, Lg: lagoon, Q: qanat, Re: reservoir, Ri: river, S: sea, Sp: spring, W: wetland.

**Table 4:** Host–parasite list of Iranian freshwater fish. The host–parasite list was organized based on the classification performed by Esmaeili et al. [81]. Host information includes current scientific name, authors’ names, authorship dates, and synonyms. It is followed by a list of parasites reported for the host categorized by higher taxon and listed alphabetically.

Host	Parasite Species
**Class Actinopterygii**	
**Order Acipenseriformes**	
**Family Acipenseridae Bonaparte, 1831**	
**Genus *Acipenser* Linnaeus, 1758**	
**Species *Acipenser gueldenstaedtii* Brandt and Ratzeburg, 1833**	*Haemogregarina acipenseris*
	*Trichodina reticulata*
	*Trypanoplasma acipenseris*
**Species *Acipenser persicus* Borodin, 1897**	*Haemogregarina acipenseris*
	*Ichthyophthirius multifiliis*
	*Trichodina reticulata*
	*Trypanoplasma acipenseris*
**Species *Acipenser stellatus* Pallas, 1771**	*Ichthyophthirius multifiliis*
	*Trichodina reticulata*
**Genus *Huso* Brandt and Ratzeburg, 1833**	
**Species *Huso huso* Linnaeus, 1758**	*Ichthyophthirius multifiliis*
**Order Cypriniformes**	
**Family Cyprinidae Rafinesque, 1815**	
**Genus *Arabibarbus* Borkenhagen, 2014**	
**Species *Arabibarbus grypus* Heckel, 1843**	
**Synonym: *Barbus grypus* Heckel, 1843**	*Ichthyobodo necator* *Ichthyophthirius multifiliis*
**Species *Barbus lacerta* Heckel, 1843**	
**Synonym: *Barbus lacerta cyri* De Filippi, 1865**	*Ichthyophthirius multifiliis*
	*Trichodina domerguei*
	*Trichodina perforate*
**Genus *Capoeta* Valenciennes, 1842**	
**Species *Capoeta aculeata* Valenciennes, 1844**	*Ichthyophthirius multifiliis*
**Species *Capoeta barroisi* Lortet, 1894**	*Ichthyophthirius multifiliis*
**Species *Capoeta capoeta* Güldenstädt, 1773**	*Arcella vulgaris* *Amphileptus branchiarum* *Chilodonella piscicola* *Ichthyobodo necator* *Ichthyophthirius multifiliis* *Stylonychia pustulata* *Tetrahymena pyriformis* *Trichodina domerguei* *Trichodina perforata*
**Comment: The subspecies, *Capoeta capoeta gracilis* Keyserling, 1861), which has been considered as an Iranian subspecies, is recognized as a full species [81]**	







**Species *Capoeta damascina* Valenciennes, 1842**	*Ichthyophthirius multifiliis*
	*Trichodina pediculus*
**Species *Capoeta razii* Jouladeh-Roudbar, Eagderi, Ghanavi and Doadrio 2017**	*Trichodina gracilis*
**Species *Capoeta trutta* Heckel, 1843**	*Ichthyophthirius multifiliis*
**Genus *Carasobarbus* Karaman, 1971**	
**Species *Carasobarbus luteus* Heckel, 1843**	*Ichthyophthirius multifiliis*
**Synonym: *Barbus luteus* Heckel, 1843**	
**Genus *Carassius* Jarocki, 1822**	*Ichthyophthirius multifiliis* *Ichthyobodo necator* *Trichodina perforata*
**Species *Carassius auratus* Linnaeus, 1758**	
**Synonym: *Carassius auratus auratus* Linnaeus, 1758**	
**Species *Carassius Carassius* Linnaeus, 1758**	*Ichthyophthirius multifiliis*
**Species *Carassius gibelio* Bloch, 1782**	*Ichthyophthirius multifiliis*
**Synonym: Carassius auratus gibelio Bloch, 1782**	
**Genus *Cyprinion* Heckel, 1843**	
**Species *Cyprinion macrostomum* Heckel, 1843**	*Ichthyophthirius multifiliis*
**Genus *Cyprinus* Linnaeus, 1758**	
**Species *Cyprinus carpio* Linnaeus, 1758**	*Goussia carpelli*
	*Ichthyophthirius multifiliis*
	*Trichodina domerguei*
	*Trichodina pediculus*
	*Trichodinella subtilis*
**Genus *Luciobarbus* Heckel, 1849**	
**Species *Luciobarbus barbulus* Heckel, 1849**	*Ichthyophthirius multifiliis*
**Synonym: *Barbus barbulus* Heckel, 1849**	
**Species *Luciobarbus brachycephalus* Kessler, 1872**	*Ichthyophthirius multifiliis*
**Synonym: *Barbus brachycephalus* Kessler, 1872**	
**Species *Luciobarbus capito* Güldenstaedt, 1773**	*Ichthyophthirius multifiliis*
**Synonym: *Barbus capito* Güldenstaedt, 1773**	*Trichodina domerguei*
	*Trichodina perforata*
**Species *Luciobarbus esocinus* Heckel, 1843**	*Ichthyophthirius multifiliis*
**Synonym: *Barbus esocinus* Heckel, 1843**	
**Genus *Mesopotamichthys* Karaman, 1971**	
**Species *Mesopotamichthys sharpeyi* Günther, 1874**	*Ichthyophthirius multifiliis*
**Synonym: *Barbus sharpeyi* Günther, 1874**	
**Genus *Schizocypris* Regan, 1914**	
**Species *Schizocypris altidorsalis* Bianco and Banarescu, 1982**	*Ichthyophthirius multifiliis*
**Comment: *Schizocypris altidorsalis* formerly identified as *Schizocypris brucei* Regan, 1914 (El-Dairi and House 2019)**	
**Genus *Schizothorax Heckel*, 1838**	
**Species *Schizothorax pelzami* Kessler, 1870**	*Ichthyophthirius multifiliis*
**Species *Schizothorax zarudnyi* Nikol’skii, 1897**	*Ichthyophthirius multifiliis*
**Family Leuciscidae Bonaparte, 1835**	
**Genus *Abramis* Cuvier, 1816**	
**Species *Abramis brama* Linnaeus, 1758**	*Ichthyophthirius multifiliis*
	*Trichodina domerguei*
	*Trichodina perforata*
**Genus *Acanthobrama* Heckel, 1843**	
**Species *Acanthobrama persidis* Coad, 1981**	*Ichthyophthirius multifiliis*
**Synonym: *Leuciscus persidis* Coad, 1981**	
**Genus *Blicca* Heckel, 1843**	
**Species *Blicca bjoerkna* Linnaeus, 1758**	*Trichodina perforata*
**Genus *Alburnoides* Jeitteles, 1861**	
**Species *Alburnoides eichwaldii* De Filippii, 1863**	*Ichthyophthirius multifiliis* *Trichodina domerguei* *Trichodina perforata*
**Synonym: *Alburnoides bipunctatus eichwaldi* De Filippi, 1863**	
**Species *Alburnus chalcoides* Güldenstaedt, 1772**	*Ichthyophthirius multifiliis* *Trypanosoma percae*
**Synonym: *Chalcalburnus chalcoides* Güldenstädt, 1772**	
**Species *Alburnus filippii* Kessler, 1877**	*Trichodina perforata*
**Species *Alburnus hohenackeri* Kessler, 1877**	*Ichthyophthirius multifiliis*
**Species *Alburnus mossulensis* Heckel, 1843**	
**Synonym: *Chalcalburnus mossulensis* Heckel, 1843**	*Ichthyophthirius multifiliis*
**Species *Alburnoides tabarestanensis***	*Ichthyophthirius multifiliis*
**Genus *Chondrostoma* Agassiz, 1832**	
**Species *Chondrostoma regium* Heckel, 1843**	*Ichthyophthirius multifiliis*
**Species *Chondrostoma orientale* Bianco and Bănărescu, 1982**	*Ichthyophthirius multifiliis*
**Species *Leuciscus vorax* Heckel, 1843**	*Ichthyophthirius multifiliis*
**Synonym: *Aspius vorax* Heckel, 1843**	*Ichthyobodo necator*
**Genus *Squalius* Bonaparte, 1837**	
**Species *Squalius cephalus* Linnaeus, 1758**	*Chilodonella cyprini*
**Synonym: *Leuciscus cephalus* Linnaeus, 1758**	*Ichthyophthirius multifiliis*
**Genus *Vimba* Fitzinger, 1873**	
**Species *Vimba vimba* Linnaeus 1758**	*Ichthyophthirius multifiliis*
**Synonym: *Vimba vimba persa* Pallas, 1814**	
**Family Xenocyprididae Günther, 1868**	
**Genus *Ctenopharyngodon* Steindachner, 1866**	*Balantidium ctenopharyngodoni* *Ichthyophthirius multifiliis*
**Species *Ctenopharyngodon idella* Valenciennes, 1844**	
**Genus *Hemiculter Bleeker*, 1860**	
**Species *Hemiculter leucisculus* Basilewsky, 1855**	*Ichthyobodo necator* *Ichthyophthirius multifiliis* *Trichodina perforata*
**Genus *Hypophthalmichthys* Bleeker, 1859**	
**Species *Hypophthalmichthys molitrix* Valenciennes, 1844**	*Chilodonella piscicola* *Cryptobia branchialis* *Goussia sinensis* *Ichthyophthirius multifiliis*



**Species *Hypophthalmichthys nobilis* Valenciennes, 1844**	*Ichthyophthirius multifiliis*
**Order Cyprinodontiformes**	
**Family Aphaniidae Hoedeman, 1949**	
**Genus *Aphanius* Nardo, 1827**	*Ichthyophthirius multifiliis*
**Species *Aphanius vladykovi* Coad, 1988**	*Ichthyobodo necator*
**Species *Aphanius sophiae* Heckel, 1847**	*Ichthyophthirius multifiliis*
**Order Esociformes**	
**Family Esocidae Rafinesque, 1815**	
**Genus *Esox* Linnaeus, 1758**	
**Species *Esox Lucius* Linnaeus, 1758**	*Ichthyophthirius multifiliis*
**Order Gasterosteiformes**	
**Family Gasterosteidae Bonaparte, 1831**	
**Genus *Gasterosteus* Linnaeus, 1758**	
**Species *Gasterosteus aculeatus* Linnaeus, 1758**	*Ichthyophthirius multifiliis*
**Order Mugiliformes**	
**Family Mugilidae Jarocki, 1822**	
**Genus *Planiliza* Whitley, 1945**	
**Species *Planiliza abu* Heckel, 1843**	
**Synonym: *Mugil abu* Heckel, 1843; *Liza abu* Heckel, 1843**	*Ichthyobodo necator* *Ichthyophthirius multifiliis*
**Genus *Chelon* Artedi, 1793**	
**Species *Chelon auratus* Risso, 1810**	*Ichthyobodo necator*
**Synonym: *Mugil auratus* Risso, 1810**	*Ichthyophthirius multifiliis*
	*Trichodina reticulata*
**Order Perciformes**	
**Family Percidae Rafinesque, 1815**	
**Genus *Perca* Linnaeus, 1758**	
**Species *Perca fluviatilis* Linnaeus, 1758**	*Trypanosoma percae*
**Genus *Sander* Oken, 1817**	
**Species *Sander lucioperca* Linnaeus, 1758**	*Trichodina perforata*
**Order Salmoniformes**	
**Family Salmonidae**	
**Genus *Oncorhynchus* Suckley, 1861**	
**Species *Oncorhynchus mykiss* Walbaum, 1792**	*Chilodonella cyprini*
**Synonym: *Salmo gairdnerii* Richardson, 1836**	*Dermocystidium salmonis*
	*Hexamita salmonis*
	*Ichthyophthirius multifiliis*
	*Trichodina truttae*
	*Tripartiella lata*
	*Trypanoplasma borelli*
**Order Siluriformes**	
**Family *Sisoridae* Bleeker, 1858**	
**Genus *Glyptothorax* Blyth, 1860**	
**Species *Glyptothorax silviae* Coad, 1981**	*Ichthyophthirius multifiliis*
**Family Siluridae Cuvier, 1816**	
**Genus *Silurus* Linnaeus, 1758**	
**Species *Silurus glanis* Linnaeus, 1758**	*Ichthyophthirius multifiliis*
	*Trichodina perforata*
**Order Synbranchiformes**	
**Family *Mastacembelidae* Swainson, 1839**	
**Genus *Mastacembelus* Scopoli, 1777**	
**Species *Mastacembelus mastacembelus* Banks and Solander, 1794**	*Ichthyophthirius multifiliis*
	*Trichodina pediculus*

**Table 5:** AUC Values of modeling freshwater fish protozoan parasite distribution under three different RCP scenarios (RCPs 2.6, 7.0, and 8.5) in two future periods (2041–2070, and 2071–2100, 10 replicated runs).

Periods		AUC_mean_	AUC_mean_ Standard Deviation
**2041–2070**	RCP2.6	0.796	0.063
	RCP7.0	0.828	0.044
	RCP8.5	0.797	0.040
**2071–2100**	RCP2.6	0.804	0.068
	RCP7.0	0.803	0.067
	RCP8.5	0.823	0.037

## Data Availability

Not applicable.
